# Aminated Graphene Oxide as a Potential New Therapy for Colorectal Cancer

**DOI:** 10.1155/2019/3738980

**Published:** 2019-03-20

**Authors:** Natalia Krasteva, Milena Keremidarska-Markova, Kamelia Hristova-Panusheva, Tonya Andreeva, Giorgio Speranza, Dayong Wang, Milena Draganova-Filipova, George Miloshev, Milena Georgieva

**Affiliations:** ^1^Institute of Biophysics and Biomedical Engineering, Bulgarian Academy of Sciences, “Acad. Georgi Bonchev”, Str., Bl. 21, Sofia 1113, Bulgaria; ^2^University of Trento, Via alla Cascata, 56/C, 38123 Povo, Trento, Italy; ^3^Medical School in Southeast University, 87 Dingjiaqiao Road, Gulou District, Nanjing 210009, China; ^4^Department of Medical Biology, Medical Faculty, Medical University – Plovdiv, Bulgaria; ^5^Technological Centre of Emergency Medicine, “Vasil Aprilov”, Blvd. 15A, Plovdiv 4000, Bulgaria; ^6^Institute of Molecular Biology “Acad. R. Tsanev”, Bulgarian Academy of Sciences, “Acad. Georgi Bonchev”, Str., Bl. 21, Sofia 1113, Bulgaria

## Abstract

Nanotechnology-based drug delivery systems for cancer therapy are the topic of interest for many researchers and scientists. Graphene oxide (GO) and its derivates are among the most extensively studied delivery systems of this type. The increased surface area, elevated loading capacity, and aptitude for surface functionalization together with the ability to induce reactive oxygen species make GO a promising tool for the development of novel anticancer therapies. Moreover, GO nanoparticles not only function as effective drug carriers but also have the potential to exert their own inhibitory effects on tumour cells. Recent results show that the functionalization of GO with different functional groups, namely, with amine groups, leads to increased reactivity of the nanoparticles. The last steers different hypotheses for the mechanisms through which this functionalization of GO could potentially lead to improved anticancer capacity. In this research, we have evaluated the potential of amine-functionalized graphene oxide nanoparticles (GO-NH_2_) as new molecules for colorectal cancer therapy. For the purpose, we have assessed the impact of aminated graphene oxide (GO) sheets on the viability of colon cancer cells, their potential to generate ROS, and their potential to influence cellular proliferation and survival. In order to elucidate their mechanism of action on the cellular systems, we have probed their genotoxic and cytostatic properties and compared them to pristine GO. Our results revealed that both GO samples (pristine and aminated) were composed of few-layer sheets with different particle sizes, zeta potential, and surface characteristics. Furthermore, we have detected increased cyto- and genotoxicity of the aminated GO nanoparticles following 24-hour exposure on Colon 26 cells. The last leads us to conclude that exposure of cancer cells to GO, namely, aminated GO, can significantly contribute to cancer cell killing by enhancing the cytotoxicity effect exerted through the induction of ROS, subsequent DNA damage, and apoptosis.

## 1. Introduction

Colorectal cancer (CRC) is the third most diagnosed cancer in men and second most frequently observed cancer in women worldwide [1, 2]. It accounts for over 9% of all cancer death and for over 63% of all cancer cases in the developed countries especially those with a Western culture [3, 4]. In the United States, colorectal cancer is the second leading cause of cancer-related deaths with less than 5-year survival rate for those with the metastatic forms of CRC [5–7]. Current approaches for treatment of metastatic CRC have only modest efficacy and are associated with significant resistance of colorectal cancer cells to chemotherapy. The need for effective treatment of metastatic CRC has driven the search for novel strategies to improve survival while minimizing toxicities and side effects in patients [8, 9]. Recently, complementary to conventional therapeutics, nanomaterial-based strategies have shown great potential in various cancer types [10, 11]. Nanomaterials as drug carriers have become a hot spot for research at the interface of nanotechnology and biomedicine because they allow efficient loading, targeted delivery, and controlled release of drugs. They are promising tools in modern therapies of cancer as they reduce the risk of side effects and multidrug resistance in cancerous cells [12, 13]. Further, nanomaterials can improve the solubility of poorly soluble drugs [14] and circulate in blood stream for longer time without being recognized by macrophages. Since the drug delivery through nanomaterials requires lower dose, it shows lower toxicity and offers increased half-life to the carried drug molecule [15].

A variety of nanomaterials, such as carbon (e.g., graphene and nanodiamond nanoparticles), some of the noble metals (gold and silver nanoparticles), organic polymers, and liposome nanoparticles, with various sizes and modifications of their surfaces have been synthesized and reported to have target-specific enhanced anticancer activity [16–21]. Among these nanomaterials, two-dimensional graphene oxide (GO) is a promising candidate for cancer treatment [22]. Graphene oxide is a single sheet of sp2 carbon atoms arranged in a honeycomb structure, containing abundant oxygen-based groups on its basal planes and its edges [23, 24]. Functional groups on its edges are hydrophilic (negatively charged carbonyl and carboxyl groups) which makes GO well dispersed in water while phenol, hydroxyl, and epoxide groups on its basal plane are hydrophobic and result in a good dispensability of GO in organic solvents [25]. Due to the presence of reactive functional groups and localized *π*-electrons at the nanosheet surface, GO nanoparticles have the ability for covalent modifications with cancer-cell targeting antibodies and molecules for use in targeted drug delivery and noncovalent interactions with aromatic antitumour drugs [26]. Besides, GO may induce the generation of reactive oxygen species (ROS) in cells, which is considered as one of the main toxicological mechanisms of various nanomaterials, including graphene [27]. Moreover, ROS generated from GO nanoparticles can alter biological macromolecules including proteins, cell membrane lipids, deoxyribonucleic acid (DNA), and ribonucleic acid (RNA) resulting in the initiation of numerous signal transduction pathways that are linked to inflammation, malignant transformation, proliferation, and apoptosis [28]. Thus, exposure of cancer cells to GO can significantly contribute to cancer cell killing by enhancing the cytotoxicity effect exerted through the induction of DNA damage. Therefore, GO not only can function as an effective drug carrier but also can potentially exert inhibitory effects on tumour cells when used by itself [23]. To improve the therapeutic effect of GO-based cancer therapy, the surface properties of GO can be modulated because it is well-known that they play a crucial role in the interaction with cells and biomolecules (particularly charge, functional groups, and C/O ratio) [25]. For example, GO nanoparticles with higher oxygen content (C/O ratio of 2.8 : 1) have been shown to contribute to oxidative stress, cytotoxicity, and pulmonary toxicity, whereas the GO with a lower oxygen content (C/O ratio of 3.1 : 1) resulted in faster immune cell infiltration, uptake, and clearance following both subcutaneous and peritoneal implantation [29].

Functionalization of graphene oxide can fundamentally change its properties and interaction with cells. For example, the modification with COOH results in passive apoptosis of T-lymphocytes while modification with PEI causes severe hemotoxicity to T-lymphocytes by inducing membrane damage [23, 30]. PEGylation from the other side can reduce the nonspecific binding of GO to biological membranes and improve its *in vivo* pharmacokinetics for better tumour targeting [31, 32]. Thus, by proper functionalization, GO can be used to design effective strategies for cancer therapy based on their increased cytotoxicity and genotoxicity.

A limited number of studies exist concerning the biological activity of aminated GO nanoparticles [33]. Correspondingly, Xu et al. [34] have shown that aminated GO induces less toxicity in macrophages than pristine GO, while Singh et al. [35] have found that GO-NH_2_ did not cause thrombotoxicity in Swiss male mice under intravenous administration. In our previous experiments, we have demonstrated that commercially available ammonia-modified GO with an approximate size of 560 nm in diameter induced apoptosis in lung cancer cells but did not influence the viability of noncancer embryonic stem cells [36]. In the present research, we have addressed the anticancer activity of aminated GO towards colorectal cancer cells using as a biological model a well-characterized mouse colon cancer cell line—Colon 26. We have evaluated the cyto- and genotoxicity potential of aminated GO nanoparticles by assessing their ability to affect cellular morphology, viability, and proliferation and their ability to induce ROS generation and apoptosis in colon cancer cells. Our results revealed that both GO samples (pristine and aminated) were composed of few-layer sheets with different particle sizes, zeta potential, and surface characteristics. Furthermore, we have detected increased cyto- and genotoxicity for the aminated GO nanoparticles following 24-hour exposure on Colon 26 cells. The last leads us to conclude that exposure of cancer cells to GO, namely, aminated GO, can significantly contribute to cancer cell killing by enhancing the cytotoxicity effect exerted through the induction of ROS, DNA damage, and apoptosis.

## 2. Materials and Methods

### 2.1. Graphene Oxide Particles

Graphene oxide (C1576, Graphenea, Spain) and ammonia-modified graphene oxide (791520, Sigma-Aldrich, Germany) are commercially available products purchased as water suspensions with a concentration of 4 mg/ml and 1 mg/ml, respectively.

For *in vitro* exposures, particle suspensions were prepared as stock solutions of 1 mg/ml in distilled water and sonicated in an ultrasonic water bath (50 Hz, UM-2, Unitra-Unima, Olsztyn, Poland) for 1 hour. The final concentrations of NPs (0.1, 1, 10, 20, and 50 *μ*g/ml, respectively) were achieved by adding the nanoparticles from the stock solution directly into the culture medium.

### 2.2. Characterization of Pristine GO and Aminated GO-NH_2_ Nanoparticles

#### 2.2.1. Transmission Electron Microscopy (TEM)

TEM micrographs of GO and GO-HN_2_ platelets were obtained by JEOL TEM (model JEM-2100, Japan), operated at 200 kV using Holey carbon film on 300 mesh nickel grids.

#### 2.2.2. X-Ray Photoemission Spectroscopy (XPS)

X-ray photoelectron spectroscopy (XPS) measurements were carried out with an Axis DLD Ultra instrument (Kratos, Manchester, UK). The spectra were recorded in constant analyser energy mode at 160 eV pass energy for survey spectra. High resolution core line spectra were performed by setting the analyser pass energy at 20 eV pass energy, and the final energy resolution was ~0.3 eV. The survey spectra were summed over 3 scans, and high resolution spectra were summed over 15 cycles. The experimental curves were fit using Gaussian components and Shirley background subtraction, with a homemade software based on the R platform (https://www.r-project.org/).

#### 2.2.3. Dynamic Light Scattering (DLS)

Dynamic light scattering (DLS) measurements were performed on Zetatrac instrument (S3500; Microtrac, Largo, FL) capable of both particle size and zeta-potential measurements. The particles' size was estimated from the distribution of velocity of the Brownian motion of GO and GO-NH_2_ nanoparticles when suspended in water [37], while the zeta potential was derived from Henry's formula for mobility where the value represents the potential difference between the dispersion medium and the stationary layer of the fluid attached to the particle.

#### 2.2.4. Atomic Force Microscopy (AFM)

AFM measurements were performed on Innova Atomic Force Microscope (Bruker Inc.) in a tapping mode in air, using standard silicon nitride (Si3N4) probe tips (tip radius < 10 nm). Samples were scanned with a rate of 0.5 Hz at 5 different locations all over the surface exploring areas of 10 × 10 *μ*m. The images (512 × 512 pixels) were captured in height and deflection modes and presented with a simple first-order flattening.

### 2.3. Cells and Cell Culture

The mouse colorectal cancer cell line, Colon 26, was obtained from the American Tissue Culture Collection (ATCC) and was maintained in DMEM medium supplemented with 10% foetal calf serum and 100 U ml^−1^ penicillin and 100 *μ*g ml^−1^ streptomycin solution at 37°C in a fully humidified atmosphere at 5% CO_2_. For routine passages, adherent cells were detached using a mixture of 0.05% trypsin and 0.02% EDTA. For cytotoxicity experiments, the confluent cells were seeded at a density of 2 × 10^4^ cells/well in a 24-well plate whereas for genotoxicity evaluation experiments, the cells were seeded in a concentration of 1 × 10^5^ cells/well in 6-well plates. Cells were cultivated at optimal conditions for 24 hours before being exposed to increasing concentrations of GO and GO-NH_2_ nanoparticles. After addition of the nanoparticles, the cells were incubated for another 24 or 48 hours then were processed according to the experiment's protocol. Control cells were processed as tested samples in the absence of nanoparticles.

### 2.4. Phase-Contrast Light and Fluorescent Microscopy

Phase-contrast light and fluorescent microscopy observations were done in order to evaluate alterations in cell morphology after 24-hour exposure to tested NPs. Phase-contrast light microscopy micrographs were taken at magnifications of 10x and 63x with a Leitz microscope equipped with a digital camera after intensive washing of cells with phosphate buffered saline (PBS, pH 7.4) with and without staining with neutral red (Sigma). For fluorescent microscopic visualization of the morphology of viable cells, the latter were stained with 0.001% fluorescein diacetate (FDA) (Sigma, Germany), dissolved in acetone for 2 min, and rinsed several times with PBS, and fluorescent micrographs were taken at magnification 10x using an inverted microscope Axiovert 25 (Carl Zeiss, Germany) equipped with a digital camera. Further, the micrographs were analysed using the ImageJ software to obtain the number of attached cells after one-day exposure to the nanoparticles.

### 2.5. Cell Counting Kit-8 Assay

Cell Counting Kit-8 (CCK-8, Sigma-Aldrich Co.) was used to evaluate cell proliferation rates after 48 hours of treatment with GO nanoparticles. It is a simple colorimetric assay based on the reduction of Dojindo's highly water-soluble tetrazolium salt, WST-8 in cells to give an orange-colour formazan dye, which is soluble in the tissue culture media. The amount of the formazan dye, generated by the activities of dehydrogenases in cells, is directly proportional to the number of living cells. The CCK-8 was performed as described previously [38]. Briefly, the cells were washed with PBS, and the CCK-8 reagent mixed with cell culture medium in ratio 10 : 1 was added to each sample. After 4 hours incubation at 37°C, at dark the amount of the coloured product of the reaction was measured spectrophotometrically at a wavelength of 450 nm.

### 2.6. DCFA-DA Analysis

Generation of hydrogen peroxide (H_2_O_2_) in Colon 26 cells was determined by using 2′,7′-dichlorofluorescin diacetate (DCFH-DA, Sigma-Aldrich). DCFH is a lipid-permeable nonfluorescent compound. The latter, when oxidized by intracellular H_2_O_2_ in the presence of cellular esterase, forms the fluorescent compound 2′,7′-dichlorofluorescein (DCF). Cells were seeded on 24-well tissue culture plates at a density of 3 × 10^4^ cells per well and were cultured for 24 hours. On the next day, GO and GO-NH_2_ particles were added in different concentrations and cells were incubated for another 24 hours. Untreated cells were used as a control. After 24 hours of treatment, the medium was replaced with a new one and 20 *μ*m of DCFH-DA was added to the cells followed by an incubation at 37°C and 5% CO_2_ for 30 min. Then the DCFH-DA containing medium was removed, the cells were rinsed with PBS, and the fluorescence intensity of DCF was detected on a spectrofluorometer with excitation at 485 nm and emission at 520 nm.

### 2.7. Single-Cell Gel Electrophoresis (SCGE)

Briefly, 1 × 10^3^ cells were mixed with 0.7% (f.c.) of low-gelling agarose (Sigma) and were layered as microgels on microscopic slides. Slides were then lysed in 146 mM NaCl, 30 mM EDTA, pH 7, 10 mM Tris-HCl, pH 7, and 0.1% N-lauroylsarcosine at 10°C for 20 min and were electrophoresed for 20 min at 0.46 V/cm. Results were visualized under a fluorescent microscope after staining of gels with SYBR green. Results were quantified by comet assay specialized software CometScore.

### 2.8. FACS Analysis

#### 2.8.1. FACS Cell Cycle Studies

FACS was performed on Colon 26 cells after incubation for 24 hours with pristine and aminated GO nanoparticles. Cells were fixed with 76% of cold ethanol immediately after incubation with GO nanoparticles and left at -20°C for 24 hours in order for the cells to be fixed. After fixation, the cells were pelleted by centrifugation, washed in PBS buffer, and treated with 100 *μ*g/ml RNAse A for 30 min at 37°C followed by staining with 50 *μ*g/ml of propidium iodide for 30 min in the dark. 50 000 cells were counted through flow cytometry, detecting red fluorescence at excitation wavelength of 488 nm. The light scattering was detected as well. The results were quantified by FlowJo V10.

#### 2.8.2. Apoptosis/Necrosis Study via Annexin V-FITC

The ability of the nanoparticles to induce cell death was evaluated by Apoptosis Detection Kit, (Annexin V—GFP-Certified Apoptosis/Necrosis detection kit, Enzo Life Sciences). Cells were spun down at 400 g for 5 min at room temperature and were carefully resuspended in 1 ml cold 1× PBS (2.68 mM KCl, 1.47 mM KH_2_PO_4_, 1.37 mM NaCl, 8 mM Na_2_HPO_4_), pH 7. Spinning down at the same conditions followed, and the pellet was resuspended in 510 *μ*l Dual Detection Reagent (500 *μ*l 1× binding buffer, 5 *μ*l Apoptosis Detection Reagent/Annexin V-Enzo Gold; 5 *μ*l Necrosis Detection Reagent). Samples were incubated at room temperature for 10 min at dark and were analysed via cytometry using 488 nm laser at FL2 and FL3 channels for apoptosis and necrosis detection, respectively. The results were quantified with FlowJo software. Two repetitions of the experiment were done.

### 2.9. Statistical Analysis

Data in this article were statistically analysed by using Students' *t*-test, where the probability levels of 0.05 were considered as statistically significant.

## 3. Results

### 3.1. Characterization of GO and GO-NH_2_ Nanoparticles

**Figure 1:**
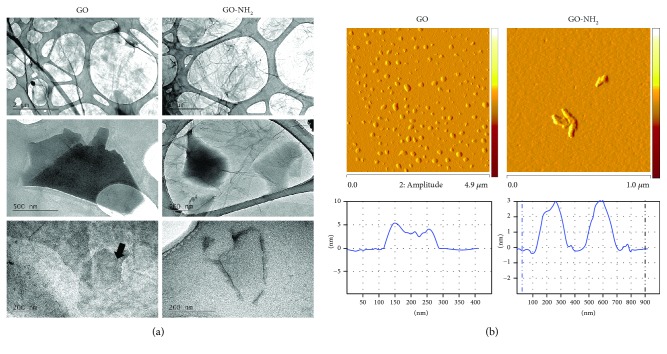
Physicochemical characteristics of GO and GO-NH_2_ nanoparticles assessed by transmission electron and atomic force microscopy. Just prior to all microscopic measurements both GO and GO-NH_2_ nanoparticles were diluted in distilled water and were sonicated for 1 hour. (a) Transmission electron microscopy of GO and GO-NH_2_ nanoparticles. (b) Representative АFM images of sonicated GO and GO-NH_2_ and the corresponding height profiles.

**Figure 2:**
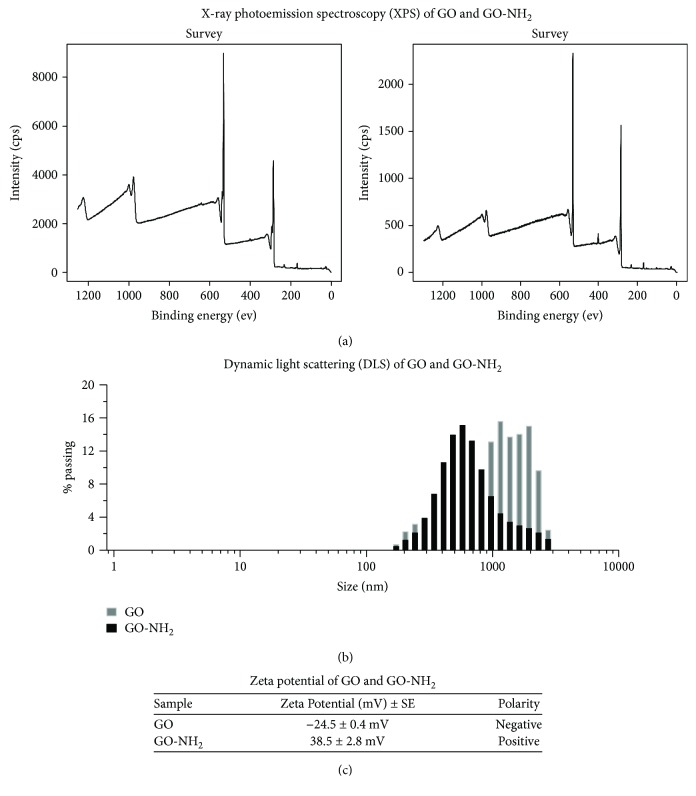
Physicochemical properties of GO and GO-NH_2_ nanoparticles assessed by X-ray photoemission spectroscopy (XPS) and dynamic light scattering (DLS). Nanoparticles were washed in buffer and after sonication were investigated by (a) X-ray photoemission spectroscopy (XPS), (b) dynamic light scattering (DLS), and (c) zeta potential analysis.

The GO nanoparticles were characterized by a number of biophysical methods including transmission electron microscopy (TEM) (model JEOL-2010, Japan), X-ray photoelectron spectroscopy (XPS, Axis DLD Ultra, Kratos, Manchester, UK), atomic force microscopy (AFM, Bruker Inc.), and nanosizer (Zetatrac instrument, S3500; Microtrac, Largo, FL). The results of the biophysical evaluation of the properties of the studied pristine and aminated GO nanoparticles are summarized in Figures 1 and 2. The aim was to determine the structure and morphology, the chemical composition of the tested GO nanoparticles, their size and thickness, size distribution, and zeta potential. Just prior to all biophysical measurements, GO and GO-NH_2_ particles were diluted in distilled water and were sonicated for 1 hour.

Figure 1(a) exhibits TEM morphology of the pristine graphene oxide and aminated graphene oxide at different magnifications (bars = 2 *μ*m, 500 nm, and 200 nm). It is obvious that GO is transparent looking like transparent thin paper structures with some folds and wrinkles. The wrinkled surface of GO provides stability and prevents collapsing back to a graphitic structure [39]. GO-NH_2_ nanoparticles have similar morphology, maintaining the two-dimensional ultrathin flexible structure, but have more corrugations and scrolling than pristine GO. The elastic corrugations and the scrolled or folded edges often result in different brightness on the surface of the GO (black arrow) [21]. Both types of GO showed the presence of mostly few-layered sheets with the tendency to scroll and wrinkle.

The AFM images of the GO and GO-NH_2_ samples (Figure 1(b)) confirmed the wrinkled 2D characteristics of the GO sheets; the images revealed that both types of GO flakes have an irregular shape as pristine GO has bigger dimensions than aminated GO (Figure 1(b), upper images). The thickness within one particle varied between that of monolayer (1-2 nm) and bilayer (3-4 nm) (Figure 1(b), the graphs below the images).

XPS analysis of GO revealed four major characteristic peaks of C1s spectrum: C–C/C=C (at 284.7 eV), C–O (at 286.2 eV), C=O (at 287.8 eV), and O–C=O (at 289.1 eV) [35] corresponding to the different functional groups on the GO sheets (Figure 2(a), left panel). GO-NH_2_ particles had the same oxygen functional groups (Figure 2(a), right panel) but with decreased peak intensities compared to pristine GO, indicating a lower oxygen content. Elemental analysis also showed that C : O ratio in pristine GO is 2 : 1 (66.36% and 33.64%, respectively), whereas in GO-NH_2_, the C : O ratio was ca. 3 : 1 (70.12% and 25.64%, resp.). The oxygen content in GO-NH_2_ was 8% lower than in GO, suggesting a partial reduction of GO during the functionalization process. The measured nitrogen content of GO-NH_2_ was ca. 3.47% while the nitrogen content in the pristine GO according to the data sheet was 0-1%. Taken together, XPS results indicate both the deoxygenation of GO and the incorporation of nitrogen in the functionalized graphene oxide nanoparticles.

Two peaks for N1s were registered in GO-NH_2_, i.e., one at 397.8 eV which is attributed to the nitrogen in C(O)N and the other at 400.3 eV which is attributed to the nitrogen in –NH_2_ [37]. The comparison of the percentages of these two kinds of nitrogen in GO-NH_2_ revealed the bonding state of the nitrogen in the GO structure. The nitrogen content of the C(O)N bonds was higher than that in GO-NH_2_ which indicated that the majority of the nitrogen was covalently attached to the GO surface [40].

Analysis of the average hydrodynamic diameter obtained from dynamic light scattering (DLS) indicated that the size of GO sheets was larger than that of GO-NH_2_ sheets and the pristine GO particles appeared more heterogeneous than that of aminated GO (Figure 2(b)). GO particles demonstrated the presence of two fractions with quite different sizes: a small fraction (9.7%) with an average particle size of 250 ± 68 nm and a main fraction (90.3%) with a particle size of 1.5 ± 0.7 *μ*m. The average particles' size of the aminated GO was 560 ± 300 nm.

Further, we have measured zeta potential of GO and GO-NH_2_ nanoparticles (Figure 2(c)). Zeta potential is an important tool for understanding the state of the nanoparticle surface and predicting the long-term stability of the nanoparticle. Nanoparticles with zeta potential values greater than +25 mV or less than -25 mV typically have high degrees of stability. Dispersions with a low zeta potential value will eventually aggregate due to Van Der Waals interparticle attractions. Results from zeta potential measurements of GO and GO-NH_2_ particles showed that GO was negatively charged in water, with a *ζ*-potential at −24.5 ± 0.4 mV, whereas GO-NH_2_ was positively charged with a *ζ*-potential at 38.5 ± 2.8 mV (Figure 2(c)). The results suggested that both nanoparticles were relatively stable in water solutions but GO-NH_2_ was more stable compared to GO, because of the higher than +25 mV value of the *ζ*-potential of GO-NH_2_, while the *ζ*-potential of pristine GO particles was around -25 mV.

### 3.2. GO and GO-NH_2_ Nanoparticles Affect the Overall Morphology and Viability of Colon 26 Cells

**Figure 3:**
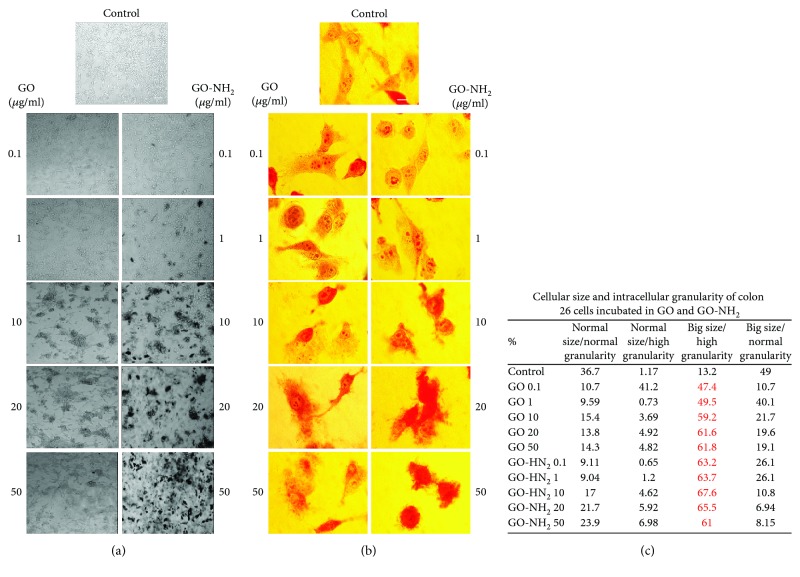
Colon 26 cellular morphology after incubation with pristine and aminated GO nanoparticles for 24 hours. (a) Phase-contrast light microscopic micrographs of Colon 26 cells incubated for 24 hours in the presence of GO and GO-NH_2_ nanoparticles at different concentrations. Magnification 10x; bar 100 *μ*m; (b) phase-contrast light microscopic micrographs of Colon 26 cells incubated for 24 hours in the presence of GO and GO-NH_2_ nanoparticles at different concentrations. Cells were stained with neutral red. Magnification 63x; bar 10 *μ*m, (c) FACS data representing relative cellular size and cell granularity of Colon 26 cells after incubation with different concentrations of pristine and aminated GO—cellular morphology according to FSC and SSC light scattering profiles of each probe. Two repetitions of the experiment are done, and results are presented as a table.

To determine whether GO and GO-NH_2_ influence cell morphology and viability, Colon 26 cells were treated with various concentrations of the two types of tested nanoparticles (from 0.1 to 50 *μ*g/ml). At the 24^th^ hour of incubation of the cells with both pristine and aminated GOs, we have evaluated the morphology of the colon cancer cells by phase-contrast light microscopy and FACS analysis following their forward (FSC) and side scattering (SSC). Representative phase-contrast micrographs of the control and treated with the nanoparticles Colon 26 cells are shown in Figure 3. The phase-contrast light microscopic observations were done at two magnifications—10x (Figure 3(a)) and 63x (Figure 3(b)). The microscopic observations under the bigger magnification were done after staining of the cells with neutral red. The logic for the use of these two magnifications in the phase-contrast light microscopy was to allow observation, first, of the overall morphology of the cell monolayers treated with GO nanoparticles (×10), while the bigger magnification (63x) and the neutral red staining of the cells was done in order to have a more detailed picture of the individual morphology of the Colon 26 cells after incubation with the nanoparticles. From the micrographs after the light microscopic observation under 10x magnification, one can very easily spot the tendency toward an enhanced, dose-dependent cell aggregation in cells exposed to pristine GO (Figure 3(a), left row images). On the contrary, cellular exposure to GO-NH_2_ did not induce such an aggregation (Figure 3(a), right row images); however, these aminated GO demonstrated a stronger adherence to the Colon 26 cells as can be concluded from the nanoparticles which were seen as remained on the cell surface even after intensive rinsing. This is probably due to the amine groups on the surface of the GO-NH_2_ nanoparticles which have high binding affinity to cellular membranes.

Staining of cells with neutral red and subsequent visualization under the light phase-contrast microscope at higher magnification (Figure 3(b)) allowed precise differentiation of the morphology of the colon cancer cells prior and after incubation with the nanoparticles. The incubation with both pristine and aminated GO nanoparticles led to abolishment of the typical cellular morphology of the studied cells. The cells got rounded and started to lose their intercellular compartment especially at higher concentrations (20 and 50 *μ*g/ml of GO and GO-NH_2_) which is evident from the micrographs. Moreover, it is very easy to be seen that this tendency was higher in cells treated with GO-NH_2_ (Figure 3(b), right panel). Additionally, in control/untreated cells, lysosomal membrane stability was observed, whereas in both GO-treated cells, the neutral red dye diffused in the cytoplasm, which was more pronounced in the aminated GO-treated cells.

FACS analyses of the relative cellular morphology after analysis of cells' FSC and SSC scattering profiles (Figure 3(c)) confirmed the above-discussed observations for the morphology of Colon 26 cells after exposure to GO nanoparticles. Cells treated with the aminated GO nanoparticles had a higher percentage of cells with big size and high cell granularity and internal complexity (see the row in which the % of cells is given in red). This population of cells with abnormal cellular morphology started to appear when the cells were treated with aminated GO at smaller concentrations (at 0.1 *μ*g/ml of GO-NH_2_ almost 60% of all cells in the population had bigger size and increased cell granularity), while this tendency was present in cells treated with pristine GO at higher concentrations of the nanoparticles, i.e., 20 *μ*g/ml and above.

**Figure 4:**
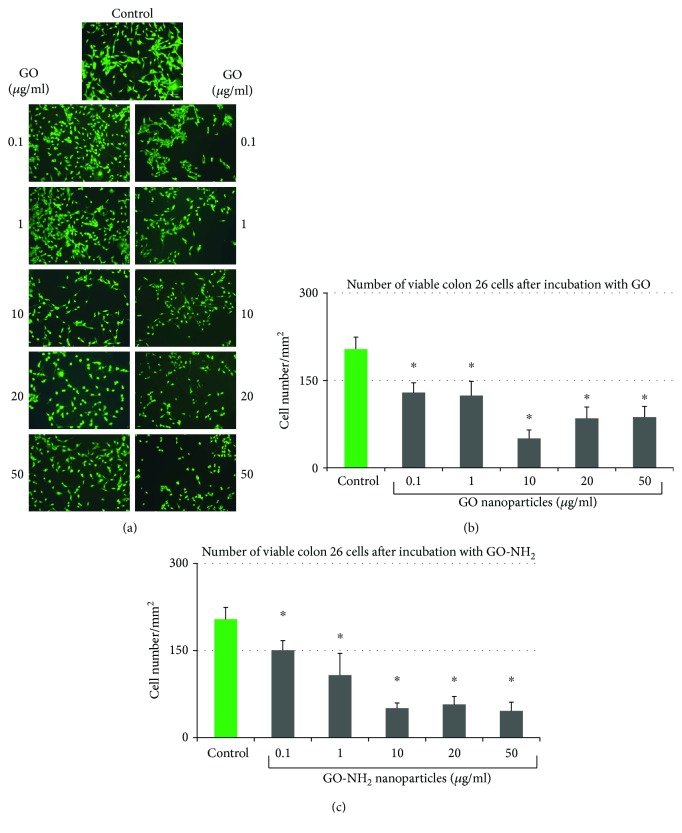
Quantitative evaluation of cellular viability of Colon 26 cells after incubation for 24 hours with pristine and aminated GO. (a) Fluorescent micrographs of FDA-stained Colon 26 cells incubated for 24 hours in the presence of GO and GO-NH2 nanoparticles at different concentrations. Magnification 10x; bar 100 *μ*m. (b) Number of viable Colon 26 cells after 24 hours of incubation in the presence of GO nanoparticles. (c) Number of viable Colon 26 cells after 24 hours of incubation in the presence of GO-NH_2_ nanoparticles (asterisks ^∗^ denote *p* < 0.05, respectively, when the tested probes are compared to untreated cells).

The FDA micrographs (Figure 4(a)) showed that the treatment with either GO or GO-NH_2_ led to a lower cellular density compared to control cells which we believe resulted from cellular aggregation, especially in GO-treated cells. The reason for this could be detachment of cells from the substrate, cell loss, and decreased cell viability, probably because of the weakened interactions with the substratum. As shown in Figure 4(a), GO and GO-NH_2_ nanoparticles altered the morphological appearance of Colon 26 cells only at the highest concentrations of 10 to 50 *μ*g/ml which was in correlation with the results from the light phase-contrast microscopic imaging of the cells (Figures 4(a) and 4(b)). Furthermore, at these concentrations, we have observed an increased number of rounded cells, i.e., cells that undergo extensive stress and are ready to die which is in accordance with the other studies of the cellular morphology after incubation with GO nanoparticles.

Quantitative evaluation of cellular viability of Colon 26 cells done by determination of the cells' number showed an inhibition of cellular survival in a dose-dependent manner in GO-NH_2_-treated samples (Figures 4(b) and 4(c)). As a matter of fact, both GO and GO-NH_2_ demonstrated their ability to reduce the viability of Colon 26 cells at the tested concentrations compared to the untreated control (Figure 4(b)), nonetheless aminated GO-NH_2_ had higher potential itself to kill the cells compared to pristine GO (Figure 4(c)). With increasing concentrations, the survival rate of Colon 26 cells treated with GO-NH_2_ decreased more sharply than that of cells treated with GO (Figure 4(c)), whereas the cytotoxic effect of GO was slightly less than that of GO-NH_2_ with one exception GO-treated cells with the highest viability loss (70%) was found at 10 *μ*g/ml which corresponded with the results with the same concentration of the aminated GO.

### 3.3. GO and GO-NH_2_ Nanoparticles Enhance ROS Production in Colon Cancer Cells

**Figure 5:**
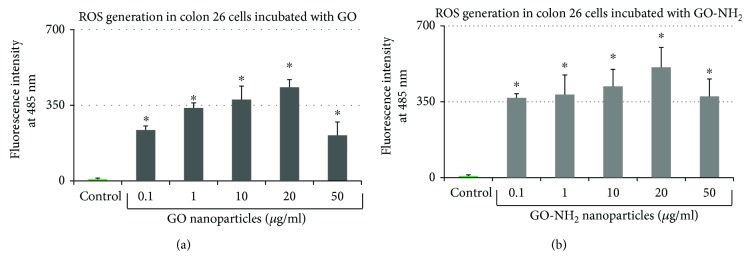
ROS production in Colon 26 cells after treatment with GO nanoparticles: (a) ROS production after 24-hour incubation in increasing concentrations of pristine GO; (b) ROS production after 24-hour incubation in increasing concentrations of aminated GO-NH_2_ (asterisks ^∗^ denote *p* < 0.05, respectively, when the tested probes are compared to untreated cells).

The ability of GO nanoparticles, both pristine and aminated, to generate ROS was determined after 24 hours of incubation with the Colon 26 cells, using the DCFH oxidation assay (Figure 5). Both types of GO nanoparticles generated statistically significant amounts of ROS production as compared to the control at all tested concentrations. As shown in Figures 5(a) and 5(b), GO and GO-NH_2_ were most efficient in generation of ROS at increasing dose up to 20 *μ*g/ml. The level of ROS generation for GO-NH_2_ at concentration of 10 *μ*g/ml was almost the same, even slightly higher than the one induced by the same concentration of GO (Figure 5(b)). Interestingly, a bell-shaped curve was observed for both types of GO (pristine and aminated) with the highest peak of ROS production generated in Colon 26 cells at concentrations of 10 – 20 *μ*g/ml of both GO and GO-NH_2_. A similar curve of ROS production has been observed previously for other carbon-based nanomaterials [41, 42]. It has been speculated that the presence of high absorbance particles such as the amorphous carbon black might cause quenching of the signal by depleting the fluorophore which in our case resulted in lesser ROS production measurement at the highest tested concentrations of GO and GO-NH_2_, i.e., 50 *μ*g/ml [43–46].

### 3.4. GO and GO-NH_2_ Treatment Influences Cell Proliferation

**Figure 6:**
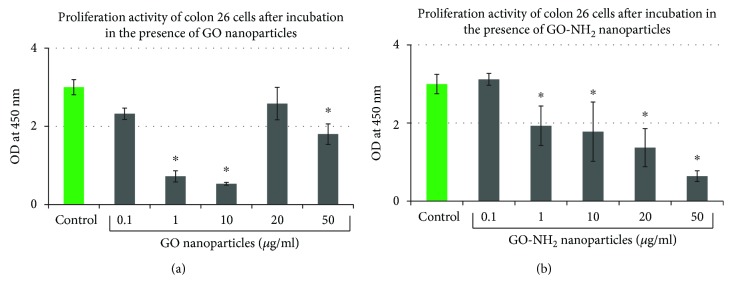
Proliferation activity of Colon 26 cells after 48 hours of cultivation of the cells in the presence of GO and GO-NH_2_ nanoparticles: (a) Colon 26 cells grown in a media supplemented with increasing concentrations of pristine GO; (b) Colon 26 cells grown in a media supplemented with increasing concentrations of aminated GO-NH_2_ (asterisks ^∗^ denote *p* < 0.05 when the tested probes are compared to untreated control cells).

Cell proliferation ability of Colon 26 cells was assessed after 48-hour exposure to increasing concentrations of GO and GO-NH_2_ nanoparticles. Overall, both types of nanoparticles (Figures 6(a) and 6(b)) exerted different effects on cellular proliferation of Colon 26 cells. A dose-dependent decrease in cell proliferation was observed in colon cancer cells treated with increasing concentrations of GO-NH_2_ particles from 0.1 to 50 *μ*g/ml (Figure 6(b)). A significant reduction in cell proliferation was measured at a concentration of 50 *μ*g/ml, which was approx. 6 times lower compared to that of the control cells (^∗^*p* < 0.05). When cells were treated with increasing concentrations of GO, an interesting result was obtained. At lower doses of GO, i.e., 1 and 10 *μ*g/ml (Figure 6(a)), the cytotoxicity of pristine GO nanoparticles was statistically greater (^∗^*p* < 0.05) than GO-NH_2_ and control cells because the cell proliferation in GO samples was significantly inhibited. After exposure, dose increased to 20 *μ*g/ml; GO induced even a slight increase in cell proliferation compared to the control. At the highest concentration of 50 *μ*g/m, a small decrease in cell proliferation rates was observed.

### 3.5. GO and GO-NH_2_ Nanoparticles Induce DNA Damage in Colon Cancer Cells

**Figure 7:**
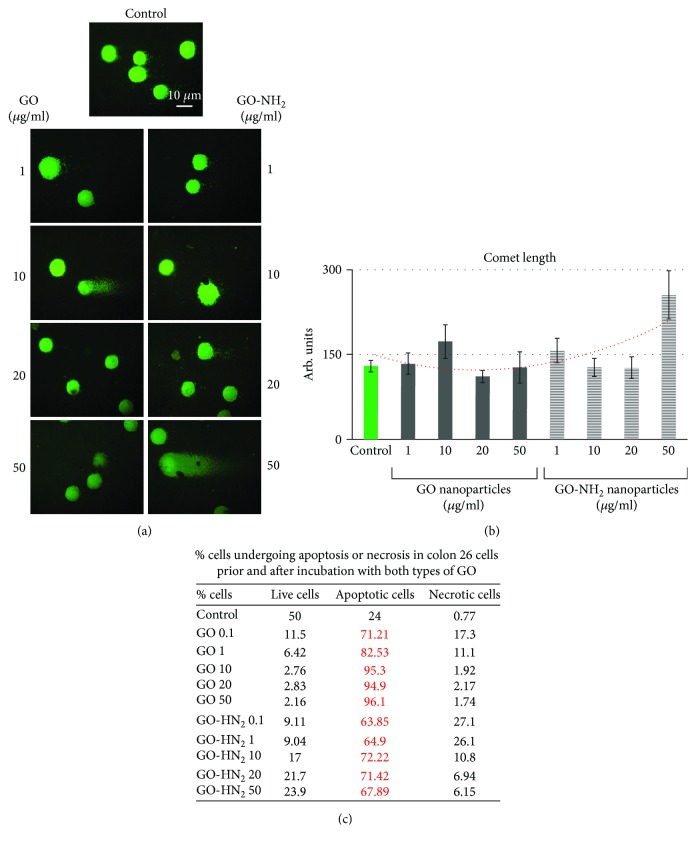
SCGE for testing the genotoxicity potential of pristine and aminated GO nanoparticles on Colon 26 cells: (a) comet images of Colon 26 cells incubated with increasing concentrations of pristine and aminated GO nanoparticles; (b) graphical representation of the parameter “Comet length” as quantified by the software CometScore. Data are represented as mean ± STDV, where *n* = 100. Additionally, the trendline is shown as a red dotted line. (c) Apoptosis/Necrosis Study *via* FACS with Annexin V-FITC kit—percentage of viable, apoptotic, and necrotic cells are displayed in the table. The % of cells undergoing apoptosis is marked in red.

In order to dissect the mechanism of cytotoxicity of the tested GO on the colorectal cancer cells, we have performed the method of comet assay, also called single-cell gel electrophoresis (SCGE). SCGE sensitively and precisely detects different damages in DNA-like single-strand DNA breaks, double-strand DNA breaks, and alkaline labile sites [47]. Colon 26 cells were treated with increasing concentrations of GO and GO-NH_2_ (1, 10, 20, and 50 *μ*g/ml) for 24 hours at optimal conditions and were subjected to SCGE. Representative images of nuclei (i.e., cells with native DNA) and comets (i.e., cells with damaged DNA) are shown on Figure 7(a). The highest used concentration of pristine and aminated GO (50 *μ*g/ml), especially that of the aminated GO nanoparticles, induced damages in DNA and led to the detection of comets as shown in the micrographs on Figure 7(a). The genotoxicity effect was not as robust as we have expected. Comet assay results were further quantified by comet assay specialized software for data analysis, CometScore, and the results are shown on Figure 7(b). “Comet Length” is a parameter in SCGE data analysis that gives representative and precise estimation of the level of genotoxicity of the tested substances. Colon 26 cells treated with pristine GO did not show the presence of any DNA damage when incubated for 24 hours with a little exception. GO at a concentration of 10 *μ*g/ml, which as in all other experiments showed higher biological activity on the cells in comparison to all other used concentrations, in the comet assay displayed the presence of 10% more DNA damage than in the control cells and those treated with the other concentrations of pristine GO used in this study. This can be seen even on the micrographs on Figure 7(a). The pristine GO led to production of comets in the tested cells at a concentration of 10 *μ*g/ml. On the contrary, the aminated GO displayed the highest genotoxicity effect on the cells at the highest used concentration of 50 *μ*g/ml. At this concentration in the Colon 26 cells, we have been able to detect comets with 50% longer comet tails than in the control cells which were an indication of severe genotoxicity induced on the cells by this concentration. The trendline on Figure 7(b) very well displays this genotoxic potential of GO-NH_2_ at the concentration of 50 *μ*g/ml and the lack of any genotoxicity at all other used concentrations of GO.

In order to investigate the mechanism of the observed genotoxicity of the tested pristine and aminated GO nanoparticles, we have performed FACS analysis with Annexin V-FITC Apoptosis/Necrosis detection kit, and the results are displayed on Figure 7(c) as a table in which the percentage of cells in apoptosis and necrosis is displayed for all tested probes. As can be easily seen (Figure 7(c): the row with % of cells in red—apoptosis), all concentrations of the tested GO nanoparticles had the potential to induce apoptosis in the studied cells. Interestingly, the pristine GO nanoparticles at concentrations of 10 up to 50 *μ*g/ml induced apoptosis in almost all cells in the probes, while the aminated GOs at these concentrations induced apoptosis in 60-70% of the cells. The last could be the reason for the observed comets in these probes while the lack of comets in pristine GO-treated cells could be a result of this higher % of cells which are already in their late stages of programmed cell death where almost all DNA is degraded and thus unable to be presented as comets.

### 3.6. GO and GO-NH_2_ Influence the Progression of Colon 26 Cells through the Phases of the Cell Cycle

**Figure 8:**
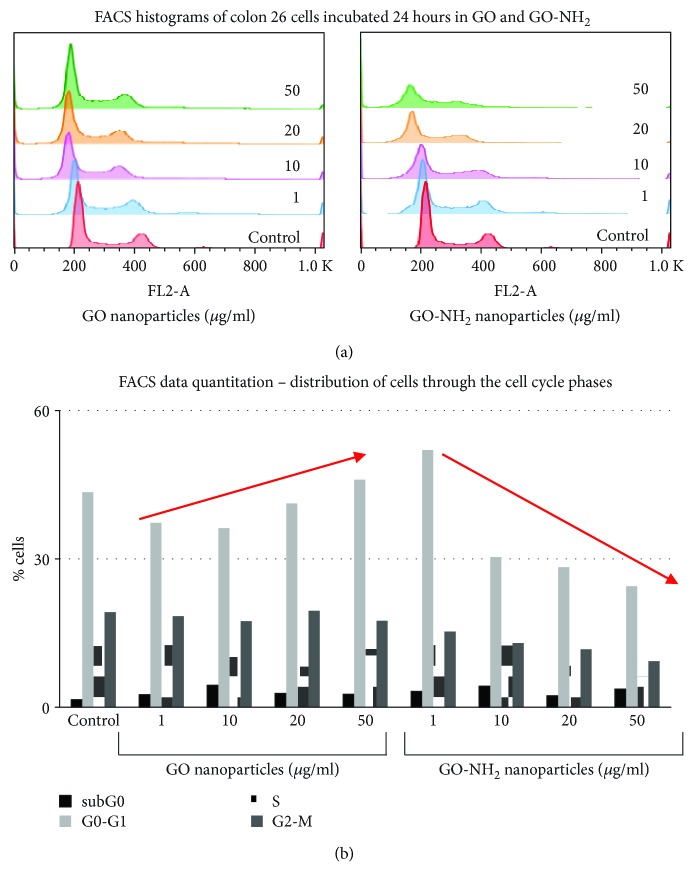
FACS analysis of Colon 26 cells cultivated for 24 hours in the presence of increasing concentrations of pristine and aminated GO nanoparticles: (a) histograms of Colon 26 cells representing the percentage of cells progressing through the phases of the cell cycle after incubation with GO and GO-NH_2_ nanoparticles; (b) representation of FACS data quantitation for colon 26 cells. Data are represented as mean, where *n* = 100 000. The red arrows show the trend in the distribution of cells in the G0-G1 phase of the cell cycle for both GO and GO-NH_2_ nanoparticles.

Any detected genotoxic activity observed at a single-cell level could potentially hurdle the progression of cells through the phases of the cell cycle. The last could lead to accumulation of mutations which often appear detrimental for the organism [48, 49]. In order to confirm our observations for the genotoxic activity of the tested pristine and aminated GO nanoparticles on the Colon 26 cells after 24 hours of incubation, we have combined the method of comet assay with the method of FACS for studying the progression of cells through the phases of the cell cycle. Cells were prepared and subjected to FACS after staining with propidium iodide. Flow cytometry analyses revealed a significant difference in the way cells were progressing through the cell cycle after incubation with pristine and aminated GO nanoparticles (Figure 8). The histograms on Figure 8(a) show the transition of cells through the phases of the cell cycle after incubation with the two types of GO nanoparticles. FACS data quantitation is represented as a graph on Figure 8(b) where 100 000 cells were measured and their characteristics were analysed by the cytometer. Figure 8(b) shows a reduction in the percentage of cells in the G0-G1 phase of the cell cycle after incubation with both types of GO with this effect being most explicitly confirmed for the aminated GO nanoparticles. The last showed a concentration-dependent reduction in the number of cells in all cell cycle phases with the most pronounced reduction in the number of cells in the phase G0-G1 at the higher concentrations (see Figure 8(b), the red arrows). These results suggest strong cytotoxic and to some extent slight cytostatic effect of the aminated GO, especially at the concentration of 50 *μ*g/ml which confirmed our comet assay results.

## 4. Discussion

Given the great interest in the identification of novel therapeutic molecules that may significantly enhance cancer cell death in a targeted and less harmful whole organism manner [50–52], the present study was designed to understand whether amination of GO ameliorates/elevates ROS production and DNA damage in colon cancer cells. This objective comes from the hypothesis that aminated GO nanoparticles have an increased toxicity towards colorectal cancer cells supported from the fact that the amine groups on the surface of the nanoparticles are robust and inexpensive ligands that ensure a high binding affinity to cancer cells and trigger different signals including those for apoptosis [53].

Our data suggest that GO-NH_2_ nanoparticles have an increased dose-dependent toxic response compared to pristine GO as understood from the results of the cell viability and proliferation assays as well as from the genotoxicity assays. Moreover, they have high potential to induce programmed cell death in colorectal cancer cells.

Analysis of the cell morphology, which is an essential sign of the physiological state of the cells and cell viability, showed a reduction in the number of viable cells under exposure to GO and GO-NH_2_ with significant alteration in overall cell morphology. Our results are in agreement with Xu et al. who have also found pronounced morphological alterations with significant cellular collapses resulting into stretching of cellular bodies in macrophages treated with pristine GO and GO-NH_2_ [34]. The dose-dependent decrease in cell density in GO-NH_2_ samples confirmed by both FDA micrographs and quantitate estimation of the number of viable cells correlates with the increased adherence of aminated GO to the colon cancer cells which is supposed to activate the apoptosis pathway in cancer cells. At the opposite, exposure to GO appears to be most cytotoxic at a concentration of 10 *μ*g/ml that might be related to the enhanced aggregation of Colon 26 cells under exposure to GO at the same concentrations. This could be also interpreted as a sign of an increased cytotoxicity because the cell aggregates have generally lower adhesiveness to the substratum resulting in cell detachment and death. Wang et al. have proposed a similar mechanism for graphene-induced cell death, in which graphene interacts with the cell surface and sends a signal that leads to downregulation, causing cells to detach, float, and finally to die [54].

Further, it was of particular interest to prove that GO and especially GO-NH_2_ nanoparticles reduced the proliferation rates of Colon 26 cells. Measurement of the rate of cell proliferation is an important prognostic indicator of colon tumour development and colorectal cancer risk. It is clearly conceivable that in colon carcinogenic process, increased or uncontrolled proliferation may lead to changes in colonic cell morphology and architecture, increased cell bulk, and heightened probability of “fixation” of any mutations that may be present [55]. It is not very clear however why GO-NH_2_ have a dose-dependent effect whereas GO nanoparticles have demonstrated maximal inhibition at concentration of 1 and 10 *μ*g/ml which corresponds to the results obtained for the number of attached cells. The mechanism of this difference of GO-NH_2_ and GO might be associated with the chemical structure of GO-NH_2_ (amine derivative of GO). The presence of the –NH_2_ groups on GO-NH_2_ surface impose the positive charge on the main surface of GO. The enhanced electrostatic interaction between positively charged GO-NH_2_ and negatively charged Colon 26 cells can be a reason for enhanced anticancer activity of GO-NH_2_ as compared to the pristine GO [56]. NP size also can affect toxicity. As an example, Akhavan et al. concluded that GO nanoplatelets with larger size have lower toxicity on MSCs (mesenchymal stem cells) than the ones with smaller sizes [57]. Oxidation state or C/O ratio is another factor influencing the compatibility of GO nanoparticles. Sydlik et al. have been previously showed that a reduction in the degree of GO oxidation from 2.8 : 1 to 3.1 : 1 results in faster immune cell in filtration, uptake, and clearance following both subcutaneous and peritoneal implantation. Aminated GO NPs used in our study have a C/O ratio of ca. 3 : 1 versus 2 : 1 C/O ratio for pristine GO. Therefore, the increase in size, charge, and C/O ratio of the tested GO pristine and aminated nanoparticles can change their effects on cancerous cell lines.

Several mechanisms are considered to be involved in the cytotoxicity induced by nanoparticles. Most nanomaterial-induced toxicity is through free-radical mechanisms, among which generation of oxidative stress mediated by ROS formation is the most important. Overproduction of ROS can induce oxidative stress, resulting in cells failing to maintain normal physiological redox-regulated functions [58]. The toxicity of GO and GO-NH_2_ NPs correlated directly to the level of intracellular ROS produced upon exposure to the nanoparticles. In general, GO-NH_2_ was more toxic for Colon 26 cells, and therefore, the measured levels of ROS were slightly higher than those in GO samples. Other authors' studies revealed that modification of GO with ammonia changed both the surface charge of the GO nanoparticles and the total acidity. In the case of the ammonia functionalization, addition of the amine functionality serves to increase the surface charge through the ammonium cation formed at physiological pH; yet, the functionalized material is also more basic than the unmodified ones because amine in nature is fairly basic (pK_a_ = 10.6) [59]. This would produce a more basic solution when compared to the pristine GO. It is a well-established phenomenon that hydrogen peroxide is more stable in acidic than basic solutions. Our data obtained with pristine and aminated GO, however, shows that both pristine and aminated GOs exert a significant degree of the breakdown of H_2_O_2_ to ROS products (i.e., hydroxyl radical). Therefore, the predominant mechanism for the production of the hydroxyl radical is in the bulk solvent (which would result from acid- or base-catalysed homolysis) but not in the interactions between the hydrogen peroxide molecules and the NP surface. This suggests that GO-NH_2_ employs their toxicity by several pathways one of which is the generation of ROS.

Generation of ROS induced by nanomaterials, directly or indirectly, plays a vital role in genotoxicity. DNA is a critical cellular target of ROS. Oxidative DNA damage involves base and sugar lesions, DNA–protein crosslinks, single- and double-strand breaks, and formation of alkali-labile sites [60, 61]. Highly reactive radicals, such as hydroxyl radicals, can damage DNA quickly in the vicinity, whereas the less-reactive ROS may interact with DNA at a distance. This complies with our results for studying the genotoxicity of the tested GOs on Colon 26 cells. Though, not as robust as we initially expected, we have been able to detect certain genotoxicity of both types of GOs (at a concentration of 10 *μ*g/ml for GO and 50 *μ*g/ml of GO-NH_2_). Analysis of the probable mechanisms of this observed genotoxicity allowed us to detect a high level of apoptosis in all tested probes, regardless treatment of cells with pristine or aminated GO nanoparticles while necrosis was at a very low rate. These results are promising for the future development of targeted therapies of colon cancer on the basis of treatment with graphene oxide nanoparticles, both pristine and aminated.

## 5. Conclusion

In summary, the present study demonstrates that aminated GO particles (GO-NH_2_) hold the potential to trigger stronger cytotoxic and genotoxic effects in the colorectal cancer cells compared to pristine GOs. The observed cytotoxicity of GO-NH_2_ in colon cancer cells involves different mechanisms like induction of ROS production, suppression of cell proliferation, elevated cytotoxicity, induction of DNA damage, and initiation of apoptosis. These effects were most pronounced at the highest concentrations pointing to a dose-dependent toxicity, whereas the most adverse effect of GO on colorectal cancer cells was under exposure to concentrations of 10 *μ*g/ml which correlated with the highest ROS production. All this suggests that both types of GO particles (pristine and aminated) exert their toxicological effect through different mechanisms. Based on these results, we have concluded that aminated GO particles have a potential to be used for the treatment of colon cancer as their effect is most predictable than those of pristine GO.

## Data Availability

All published data are freely available for academic and research use.
